# Creating a collaborative peer writing group during residency

**DOI:** 10.1080/10872981.2018.1563421

**Published:** 2018-12-31

**Authors:** Heather Lukolyo, Elizabeth M. Keating, Chris A. Rees

**Affiliations:** a Department of Pediatrics, Baylor College of Medicine, Houston, TX, USA; b Department of Pediatric Emergency Medicine, University of Utah, Salt Lake City, UT, USA; c Division of Emergency Medicine, Boston Children’s Hospital, Harvard Medical School, Boston, MA, USA

**Keywords:** Residency, tips, research, peer writing group, collaboration

## Abstract

Residency training principally aims to educate trainees on the clinical care of patients. This arduous period of training also offers access to mentorship and institutional resources for research; however, the demands of rigorous clinical training present barriers to conducting meaningful research during this time. Peer writing groups have been shown to be effective means to increase scholarly output among faculty but have not been well described as models to increase scholarly output among residents. The authors established a collaborative peer writing group during residency that resulted in several peer-reviewed publications along with national and international conference presentations. Based on their experience and a review of the literature, the authors present practical tips on establishing and implementing a resident peer writing group.

## Introduction

Participation in scholarly activity during residency training is required by the Accreditation Council for Graduate Medical Education [1] and synthesizing medical literature is central to a physician’s ability to deliver high-quality care. While dissemination through publication in peer-reviewed journals and presenting scholarly work at professional meetings is important, there are many barriers to transforming scholarship into this type of scholarly output during the intense and demanding clinical training period of residency. Such barriers include variable quality of formal instruction on research as well as lack of both time and invested mentors [2–4]. In addition, residents often do not understand the clinical relevance of research, and get lost in the processes of conducting research [5]. Furthermore, one study suggested that resident participation in research may result in decreased clinical exposure [6]. Many have published descriptions of formal programs within residency ranging from dedicated research blocks to longitudinal research curricula to promote resident scholarship [7–10] and have shown that residents can indeed conduct meaningful research despite the clinical demands of residency [3,11].

Peer writing groups, in which colleagues work together on one or more endeavors to increase the collective scholarly output, assist in overcoming barriers such as perceived lack of writing skills, low self-efficacy, inertia, and writer’s block [12]. While peer writing groups have been described as a mechanism to increase scholarly output (i.e., published, peer-reviewed articles) among faculty members [13,14], this model has not been well described as a means to promote such output among residents. We present our experience from residency as a collaborative peer writing group outside the requirements of a formal residency program as a way that self-motivated residents can increase scholarly output.

To be successful, we posit that a peer writing group must address the three core psychological needs of individuals as described by self-determination theory – autonomy, competence, and relatedness – to drive the intrinsic motivation of each member [15]. Autonomy relates to the feeling that one is completing a task by one’s own volition, rather than because of a requirement; competence refers to feeling capable of completing the task; and relatedness refers to the need to feel a sense of belonging, or connectedness, to others [15]. Through our model, we collaborated on scholarly projects during residency that led to publication of five peer-reviewed manuscripts [16–20], one accepted peer-reviewed publication, presentation of eight abstracts at national and international conferences, and submission of one additional manuscript currently under review. The practical tips below outline how to establish, implement, and leverage success from a resident peer writing group, integrating evidence from the literature and our own group’s experiences.

The tips address the barriers to publication in the context of residency specifically, as this is an area that has not been widely reported on. Rather than focusing on residents’ learning processes in conducting scientific research, this article offers practical tips for residents to be scholarly productive in order to further scientific understanding through dissemination of research in peer-reviewed journals. Further, scholarly output is beneficial for the future career of residents. This article will be useful for residents who wish to write and publish scientific articles during residency, and will also be of interest to medical educators who mentor residents with research interests.

After collaborating on several projects, we received feedback from multiple mentors that our approach to scholarship during residency was unique, which prompted us to think about the elements of our peer writing group that made it successful. We conducted an initial literature review to understand models of peer writing groups and to identify barriers to research in residency. In so doing, we found that the peer writing group model in the context of residency is not well described in the literature. We then iteratively created tips for pursuing scholarship during residency and conducted further review to identify supporting literature to for each tip.

### Identify motivated co-residents with shared interests

When establishing a resident peer writing group, identifying colleagues with shared research and career interests is essential. They do not need to be experienced researchers, as one of the goals of the group is to learn by doing, and from one another. As much of the work within a resident peer writing group is self-directed, the ideal member should be intellectually curious and intrinsically motivated to improve their research and writing skills. Intrinsic motivation involves doing something because it is ‘inherently interesting or enjoyable’ rather than because of external factors [15], such as residency program requirements. Motivating factors vary but may include a desire to enhance one’s publication record for fellowship or job applications, develop a research portfolio, or strengthen scientific writing and research skills. Peer writing groups can take the form of collaborative writing groups, where writing tasks are shared among group members on a joint project; an accountability group that functions to hold members responsible as they work on individual projects; or a co-located writing group in which members come together to write in a shared physical space while working on individual projects [21]. In our experience, the collaborative writing group model worked best as residents to maximize learning and shift responsibilities when alternating members were on particularly demanding rotations.

### Leverage resources of residency program

The Accreditation Council for Graduate Medical Education states that ‘residents should participate in scholarly activity’ [1], but the broad nature of this statement allows residency programs flexibility in its interpretation. In order to meet this requirement, residency programs offer various resources to residents, including funds for research or to attend scientific conferences, faculty mentors, statisticians, protected research time, research directors, specific research tracks, or a day in which residents present their research to colleagues and faculty [4,11,22,23]. Institutions have different resources available for residents interested in research, so we advise that residents speak with their program director or assigned mentor(s) to identify what their specific program offers.

Furthermore, investigators at some institutions may have ongoing research projects that would benefit from including a motivated resident. Program directors or assigned mentors can point trainees toward ongoing research endeavors. In addition, when the peer writing group generates a research idea independently, we suggest capitalizing on residency program resources to pursue the project. For example, our peer writing group came up with an idea for a survey study, and then sought out faculty mentors with expertise in survey design methodology at our institution.

In addition, with all members of a resident peer writing group looking for ongoing opportunities, more possibilities for engagement in research arise. When they do, other members of the group may be included if the principal investigator agrees and there are roles for the other members. For instance, if one peer writing group member is invited to participate in a project, they in turn can invite the other members to participate.

### Seek mentorship

Mentorship is a key component in successful research during residency. Studies have shown that increased faculty mentorship leads to increased resident scholarly output [4]. In addition, lack of mentorship support has been cited as a significant barrier to successful resident scholarly activity [10].

Outstanding mentorship was an important element to our success as a resident peer writing group. We had mentors who supported our efforts and provided rapid feedback on our projects. As seasoned researchers themselves, mentors are able to build trainees’ competence, in line with the self-determination theory, by supporting residents in completing research tasks. For instance, our mentors provided guidance toward successful publication by providing technical expertise and by helping us refine research questions, design research protocols, and improve scientific writing skills.

We suggest identifying mentors in your area of interest and browsing their institutional websites and previous publications to see what their research interest areas are. Start where your research areas align and meet with multiple potential mentors to see who you could envision working with.

### Utilize peer learning and one another’s strengths

A resident peer writing group fosters a supportive environment for learning and collaboration. Previous studies indicate the positive impact of a supportive environment for academic writing [13,14]. Peer learning can be defined as ‘a collaborative and cooperative teaching and learning strategy [in which] learners are active equal partners, … self-directed, … and actively participate in discussions and feedback’ [24]. Peers should facilitate learning by providing emotional support and assisting each other with tasks. Moreover, the supportive environment also enhances the feeling of relatedness, which drives the internal motivation and commitment of the members [15]. The resident peer writing group presents an opportunity to learn through doing in a ‘community of practice’ in which members come together to pursue a shared enterprise, and gradually master skills through active engagement [25].

Working in a resident peer writing group also enables collaboration between members with varied backgrounds, skillsets, and interests. For instance, if one group member excels at quantitative analysis, while another member excels at qualitative research, these individuals can complete tasks related to their area of expertise and also teach other group members about the area. Members may also possess varied skill levels in organization and task management, and the group should also leverage these skills. Members of our resident peer writing group naturally gravitated toward contributing their strengths. Furthermore, we benefited from the social pressure of completing assigned tasks.

### Generate research ideas

A resident peer writing group can implement a think-tank approach to generate research ideas based on members’ research interests and through identification of gaps in the literature. Because the resident peer writing group is structured outside the margins of residency program requirements, members can exercise autonomy in selection of research topics. We recommend taking a broad view of scholarly inquiry as described by Boyer that encompasses discovery, integration, application, and teaching [26]. Briefly, discovery involves investigation of the unknown; integration involves synthesizing, contextualizing, and interpreting research into larger intellectual patterns; application involves the use of knowledge to address problems; and teaching involves skill development and dissemination of knowledge. Opportunities to investigate research questions are varied and include chart reviews, cross-sectional surveys, needs assessments, and systematic reviews, to name a few. Our resident peer writing group formed around a shared interest in global health, and we collaboratively developed research questions about medical education, capacity building for research in low- and middle-income countries, and pediatric HIV-associated malignancies.

### Assign equitable authorship

Authorship and authorship order should be determined at the outset of any research project in concert with all members of the peer writing group and the mentor. As a successful resident peer writing group publishes more articles, challenges in assigning authorship and authorship order may arise. In fact, one study reported that as many as 36% of researchers reported having had disagreements about the assignment of authorship [27]. To reduce disputes over authorship, some have suggested alternating the collaborator who is listed as first author in research originating from one data set or series of projects [28]. Authorship order should be based on the amount of work each collaborator will contribute to the project, with the first author traditionally taking the lead in ensuring that each step of the project is moving forward and, ultimately, drafting and submitting the final manuscript. Another approach well suited for resident peer writing groups is for the lead author to write the abstract and discussion, with others contributing to the introduction, methods, and results. Our peer writing group occasionally deviated from traditional role assignments when necessary to increase scholarly output. For instance, in the first articles we published together, the second and third authors took more of an active role in manuscript writing. Additionally, our resident peer writing group assigned some tasks such as data abstraction to second and third authors, allowing projects to move forward without funding for support staff.

Our resident peer writing group kept a table with authorship assignments for each project with accompanying roles and duties that merited each authorship position. We found this to be a helpful method of reminding each contributor of their roles and to ensure that members rotated taking the lead first author role, as well as second and third authorship positions, on various projects. Once agreed upon, authorship order should not be changed without critical review by each member of the peer writing group. Such undiscussed changes can create resentment and preclude collaboration on future projects.

### Define realistic goals with definitive endpoints

Successful academic pursuits amidst a demanding clinical schedule in residency will only result with *feasible* research endeavors. Setting small, realistic goals at each step of the research process (i.e., target dates for application to institutional review board, data acquisition and analysis, writing, revisions, and submission) facilitate research within a resident peer writing group. Realistic research goals in residency should be flexible and should be made thoughtfully, understanding that some rotations are busier than others.

Keeping the importance of flexible timeframes during residency in mind, it is imperative that resident peer writing groups agree on goals and timelines. In academic medicine, the moniker is that ‘a project is not completed until the article is published’. Seeing projects to manuscript publication is an important and feasible goal in resident peer writing groups, despite time constraints inherent in rigorous clinical training. Our group found that setting internal deadlines for completion of key tasks helped us stay on track to meet the larger goal of manuscript publication.

### Create project timelines

A previous description of a resident research project emphasized the importance of having a set study date (i.e., the Boston Marathon) in the successful implementation of a resident-driven research project [29]. However, most research projects do not have a definitive event before which many steps need to be taken. As such, resident peer writing groups should establish internal timelines to keep other members on track. Perhaps more important than setting these timelines is keeping each other on track while balancing the demands of busy clinical schedules. Residents in the peer writing group should hold one another, as well as their faculty mentors, accountable to timelines to ensure project completion.

Our resident peer writing group employed a modification of Edwards’ ‘short stops’, or periods of time set aside for meetings every four to six weeks to meet specifically to discuss the progress on each project [30]. We discussed the status of each concurrent project and ensured action was being taken to advance each step of the research process from defining research questions to manuscript or abstract submission. These frequent meetings helped each collaborator prepare before the session and ask for specific input from other members of the resident peer writing group. Each meeting ended with all members assuming specific responsibilities for the next steps along with defined deadlines for each task.

### Take turns carrying the load

One way for a resident peer writing group to stay on track with multiple ongoing projects is taking turns carrying the work load. Residents complete many busy rotations that do not allow for a significant amount of scholarly work outside of the hospital, such as intensive care unit rotations or busy ward rotations. In our resident peer writing group, when one collaborator was on a clinically heavy month, the other two collaborators took a heavier research work load, or sometimes even moved projects forward without the busier colleague, understanding that they would do the same when others were on time-intensive rotations. This way, rather than a busy rotation halting projects completely, one less busy collaborator could serve as the temporary ‘driver’ of our projects.

Some residency programs offer research rotations to increase resident participation in research [7]. Such dedicated research rotations may lead to increased scholarly output during residency [10,31]. Our program provided a research rotation during the second year of residency, as well as a second research elective one could participate in during one’s third year. We recommend assigning heavier research workloads to residents during their dedicated research rotations to capitalize on that dedicated time. Our peer writing group did this, resulting in several months throughout residency where at least one of us had dedicated time to advancing our projects.

### Leverage early successes to continue momentum

As the resident peer writing group gains experience in conducting research, the group will likely gain academic momentum while earning the respect of mentors. With early demonstrated success, it becomes easier to identify additional mentors and research projects in which to become involved. With each success comes the potential for further collaboration; however, the group should choose future projects wisely to avoid overcommitting or sacrificing clinical learning. As the resident peer writing group becomes more skilled, they are better able to generate feasible projects and implement them during residency. As each group member gains confidence in their research skills, they are able to more effectively lead the team. One study found that publication of one article during residency was predictive of future manuscript submissions as a resident, demonstrating academic momentum [11]. Once we had successfully published our first collaborative article, our resident peer writing group found it was easier to tackle new projects and that faculty mentors were more eager to work with us given a proven track record.

### Maintain collegiality and open communication

Open communication contributes to the sense of relatedness that drives the inherent motivation and ultimately the success of a resident peer writing group. The group climate should be warm, collaborative, and promote free expression of emotions, questions, and doubts, thus satisfying the relatedness component of social-determination theory [15]. Our group used multiple channels to communicate about progress on projects, including in-person meetings, conference calls, emails, and a group text.

Recognizing time constraints in a resident’s schedule, many of these meetings were held after-hours over dinner or in each other’s homes. Such settings helped create a creative and productive space outside of the hospital walls and fostered collegiality and relatedness. Though meeting in person is preferred for ‘short stops’, when we were not able to meet in person, we sent emails listing each project, the current status, and next steps including roles and steps each collaborator needed to fulfill. In cases where we did not meet in person, one collaborator was assigned the role of sending a follow-up email to delineate roles and set deadlines.

It is important to discuss and reflect on previous successes and accomplishments. Additionally, just as in any research group, there are bound to be disagreements and misunderstandings, which must be discussed so that no group member harbors resentment. The ultimate determinant of whether a peer writing group is successful is trust among its members; focusing not on who gets credit, but on the overall goal of advancement of science. Each member must feel that the others care for him or her, and ultimately focus on the needs of each other.

### Lay the foundation for ongoing collaboration

Having multiple articles published through a peer writing group by the time of residency graduation is an asset to graduates as they begin the next step of their medical career, whether that is fellowship training or a faculty position. In addition, the peer writing group opens doors for future collaboration. Even if group members follow divergent career trajectories following residency, they may continue to use the foundation built in residency for new projects throughout their careers. Leveraging the foundation established during residency, our peer writing group continues to collaborate on projects of mutual interest, despite each member moving on to different institutions and roles.

## Conclusions

A resident peer writing group may help residents capitalize on peer-learning, respectful collaboration, group momentum, and the intellectual curiosity and strengths of their peers to drive scientific inquiry and engagement in the process of scholarship, ultimately increasing scholarly output during residency. Our collaborative resident peer writing model addresses the psychological needs of autonomy, competence, and relatedness as described in self-determination theory to foster the internal motivation that drives scholarship. These practical tips offer advice for motivated residents to establish a collaborative resident peer writing group and leverage successes from this group during residency.
